# The Journey of *in vivo* Virus Engineered Dendritic Cells From Bench to Bedside: A Bumpy Road

**DOI:** 10.3389/fimmu.2018.02052

**Published:** 2018-09-11

**Authors:** Cleo Goyvaerts, Karine Breckpot

**Affiliations:** Laboratory of Molecular and Cellular Therapy, Department of Biomedical Sciences, Vrije Universiteit Brussel, Jette, Belgium

**Keywords:** viral vaccine, dendritic cell, T cell, cancer, immunotherapy, preclinical and clinical

## Abstract

Dendritic cells (DCs) are recognized as highly potent antigen-presenting cells that are able to stimulate cytotoxic T lymphocyte (CTL) responses with antitumor activity. Consequently, DCs have been explored as cellular vaccines in cancer immunotherapy. To that end, DCs are modified with tumor antigens to enable presentation of antigen-derived peptides to CTLs. In this review we discuss the use of viral vectors for *in situ* modification of DCs, focusing on their clinical applications as anticancer vaccines. Among the viral vectors discussed are those derived from viruses belonging to the families of the *Poxviridae, Adenoviridae, Retroviridae, Togaviridae, Paramyxoviridae*, and *Rhabdoviridae*. We will further shed light on how the combination of viral vector-based vaccination with T-cell supporting strategies will bring this strategy to the next level.

## Dendritic cells: nature's adjuvant

Since their discovery in 1973, it was clear that dendritic cells (DCs) stood out above the immune cell pack (1, 2). They are morphologically distinct from all other immune cell types and are gifted with an unparalleled capacity to take up, process and present self and foreign antigens to both CD4^+^ and CD8^+^ T cells. DCs are critical intermediaries between the innate and adaptive immune systems, as they stimulate, regulate, and shape both immunity and tolerance in all its disguises. Ralph Steinmann, who discovered these cells, was awarded the Nobel Prize for Medicine in 2011, because the discovery of DCs changed medicine (3).

Dendritic cells in both humans and mice represent a population of at least four different subtypes with distinct phenotypical and functional characteristics (4–7). These subsets are: plasmacytoid DCs (pDCs), two subsets of conventional DCs (cDC1 and cDC2), and inflammatory DCs. The latter represent a monocyte-derived subset that appears during inflammatory responses (Table 1). Recently, additional types of human blood DCs, monocytes, and progenitors were revealed using single cell RNA-sequencing. The group of Prof. Nir Hacohen identified pDCs next to cDC progenitor-derived cDC1 (Clec9A^+^) and two types of CD1c^+^ cDC2, of which one can also be derived from CD14^+^ DCs. Furthermore they found a CD141^−^ CD1c^−^ CD11c^+^ DC subset derived from CD16^+^ monocytes and an AXL^+^ Siglec6^+^ subset (8). Future research will have to unravel a possible murine representative for the human cDC2 and AXL^+^ Siglec6^+^ DC subset. Also, Langerhans cells have been considered an important DC subset for vaccination as they are localized in the epidermis (HLA-DR^+^ CD11c^+^ CD1a^+^ CD207^+^). However, recent evidence suggests that they are related to macrophages, another antigen-presenting cell (APC) type with potential antitumor activity (9).

**Table 1:** Overview of currently described murine dendritic cell subsets with their human counterparts.

	**cDC1**	**cDC2**	**pDC**	**Infl DCs**
	** 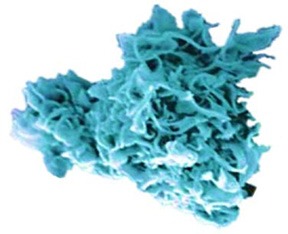 **	** 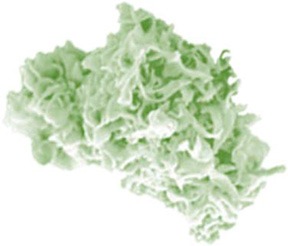 **	** 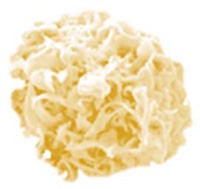 **	** 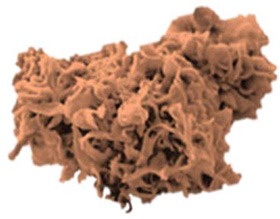 **
**Mouse**			
** Common name**	**CD8**α^+^ **cDC (LT) CD103**^+^ **cDC (NLT)**	**CD4**^+^ **CD11b**^+^ **cDC (LT) CD11b**^+^ **cDC (NLT)**	**SiglecH**^+^ **BST2**^+^ **pDC**	**Ly6C**^+^ **monocyte derived infl DCs**
** Other markers**	TLR3^+^ CADM1^+^ XCR1^+^ BATF3^+^ CLEC9A^+^ FLT3^+^ CD205^+^	CD24+,SIRPα^+^ CD11c^+^ FLT3^+^	B220^+^ Ly6C+ TLR7^hi^ TLR9^hi^	FcεRI^+^ CD11b^+^ CD206^+^ CD115^+^ CD64^+^ DC-SIGN^+^ MAC-3^+^
**Human**			
** Common name**	**CD141**^+^**(BDCA-3) cDC**	**CD1c**^+^**(BDCA-1) cDC**	**CD123**^+^ **pDC**	**CD14**^+^ **monocyte derived infl DCs**
** Other markers**	TLR3^+^ CADM1^+^XCR1^+^ FLT3^+^ CLEC9A^+^ CD162^hi^ CD205^hi^	SIRPα^+^ CD11b^lo/+^ FLT3^+^ CD11c^+^	CD45RA+,BDCA-2^+^, BDCA-4^+^ TLR7^hi^ TLR9^hi^	FcεRI^+^ CD11b^+^ CD206^+^ CD115^+^ CD64^+^ BDCA-1^+^ CD1a^+^ CD172a^+^ DC-SIGN^+^ CD1c^+^
**Conserved**			
** Phenotype**	**TLR3**^+^ **CADM1**^+^ **XCR1**^+^ **CLEC9A**^+^	**CD1c**^+^ **SIRP**α^+^ **CD11b**^+^	**TLR7**^hi^ **TLR9**^hi^	**Fc**ε**RI**^+^ **CD11b**^+^ **CD206**^+^ **CD115**^+^ **CD64**^+^ **DC-SIGN**^+^ **ZBTB46**^+^
** Functions**	Cross-presentation IL-12 secretion T_H_1/2 polarization TLR3-induced IFN-λ production	Presentation to CD4^+^ T cells IL-1β, IL-6, and IL-23 production T_H_2 and T_H_17 polarization	Viral sentinels TLR7/9-induced IFN-α/β and IFN-λ production	Highly adaptable with amongst others IL-12 or IL-23 secretion + **TipDCs** = TNFα and iNOS producing subset of infl DCs

*General hallmarks not included in this table are MHCII^hi^ and CD11c^+^, LT, lymphoid tissue; NLT, non-lymphoid tissue*.

Different DC subsets are endowed with distinct functions. pDCs are specialized in sensing viral infections. To that end, pDCs use toll-like receptor 7 (TLR7), TLR9 and stimulator of interferon genes (STING) for sensing of nucleic acids (ssRNA, dsDNA, and cytosolic DNA, respectively). Triggering these receptors results in the production of high levels of type I interferon (IFN) (10). A key function of cDC1 that requires the production of IL-12 and/or type I IFN, is activation of cytotoxic CD8^+^ T lymphocytes (CTLs) via cross-presentation of antigens and linked herewith stimulation of CD4^+^ T helper 1 (T_H_1) responses (11–14). cDC1 selectively express TLR3 enabling them to sense dsRNA, and similar to pDCs, cDC1 express TLR9 for sensing of dsDNA (15). The expression of TLR3 and TLR9 explains the cDC1s' ability to produce type I IFN. cDC2 and inflammatory DCs are also able to produce IL-12, stimulate CD4^+^ T_H_ cells and CD8^+^ T cells by cross-presentation. Depending on their activation, they will instigate a specific immune response. Both cDC2 and inflammatory DCs are equipped with a wide range of TLRs allowing them to become activated upon contact with various stimuli like polyI:C (TLR3), LPS (TLR4), and R848 (TLR8) (15, 16). The DC subsets co-operate in a wide range of immune responses, through mechanisms that are relatively conserved across mammalian species. The knowledge that human DC subsets have counterparts in mice enables the use of murine models to study the potential of DCs for cancer vaccination.

In general, antitumor vaccines comprise one or more tumor-associated antigens (TAAs) and an adjuvant to avoid induction of TAA-specific tolerance. Due to the exquisite capacity of DCs to cross-present and stimulate antitumor immunity, they have been applied as nature's adjuvant in cancer vaccination studies. Therefore, autologous DCs are generally loaded *ex vivo* with one or more TAAs, possibly with additional DC activating stimuli. Subsequently, they are transferred back to the patient to induce a TAA-specific CTL response. To exemplify, Sipuleucel-T, trade name Provenge (Dendreon), was the first autologous DC-vaccine that was approved by the FDA in 2010. More specifically it was approved for the treatment of metastatic, hormone-refractory prostate cancer. This vaccine consisted of autologous DCs that were loaded with a fusion protein consisting of prostatic acid phosphatase (PAP) and granulocyte macrophage-colony stimulating factor (GM-CSF) (17).

In most clinical trials with DC-based vaccines, autologous monocyte-derived DCs (moDCs) are used (18). However, these moDCs do not recapitulate the natural diversity of DCs, but rather mimic inflammatory DCs. The awareness that moDCs might not be ideally suited for vaccination purposes together with their overall limited efficacy in clinical trials, has stimulated research in the use of cDCs or pDCs in the clinic (19, 20). Comparing clinical trials is a challenging task, as there are significant differences in (i) type of antigens used, (ii) type of system used to deliver the antigens, (iii) protocol used to activate the DCs, (iv) route of DC administration, and (v) heterogeneity of inclusion criteria with patient selection bias. Nonetheless, we dare to state that clinical data do not hint at a better outcome upon cDC- or pDC-based cancer vaccination compared to the clinical data obtained with moDC-based vaccines (21–23). This could suggest a need for cooperation between multiple APC subsets to induce effective antitumor immunity (24, 25). When optimal priming of antiviral CD8^+^ T cells was investigated, a response fundamentally similar to an antitumor immune response, accumulation of pDCs at sites of CD8^+^ T cell activation led to local recruitment of cDC1 via XCL1 chemokine secretion by the CD8^+^ T cells. The CD8^+^ T cell-mediated reorganization of the local DC network allowed the cooperation of cDC1 and pDCs, and enhanced the maturation and subsequent cross-presentation of antigens by cDC1 (26). These findings suggest that stimulation of only one DC subset is most likely not optimal for CTL stimulation. Together with the fact that vaccination with patient-specific, *ex vivo* engineered DCs is a very costly and cumbersome method (27–30), research moved to the *in situ* engineering of DCs. This allows targeting of natural DC subsets. Moreover, it implies an assent for cooperation with other subsets and as such optimal CTL activation *in situ* (24).

We can roughly distinguish four types of *in situ* DC-directed vaccines: naked proteins, naked nucleic acids, viral vectors and nanoparticles (25, 31–34). In general, naked protein- and nucleic acid-based vaccines are relatively easy to generate. However, they need to be co-delivered with an adjuvant to achieve robust antitumor immunity. In contrast, nanoparticles and viral vectors represent more immunogenic vaccines. For viral vectors, this is explained by the fact that TAAs are truly produced by the viral vectors upon infection next to the delivery of intrinsically immunogenic viral proteins that trigger a type I IFN response (35–37). When *in vivo* vaccination of mice with a viral vector was compared to peptide, DNA, or DC-vaccination, the strongest tumor-specific immune responses were elicited with viral vectors (38–40).

Despite this knowledge, viral vectors have not taken the lead in clinical antitumor vaccination trials. Therefore, we review the use, advantages as well as shortcomings of viral vector vaccines, highlighting their potential. In particular, we focus on their clinical application. Furthermore, we touch upon pre-clinical data for the viral vector types that have not been clinically tested yet.

## Viral anticancer vaccines that have entered the clinical arena: from bench to bedside

Antitumor vaccination strategies using viral vectors can be subdivided into two main classes. The first class comprises viral vectors that encode TAAs to engineer tumor-specific DCs *in situ*. The second class consists of non-replicating apoptosis-inducing vectors or oncolytic viruses that are used to induce tumor cell death, and as such stimulate local and systemic immunity toward released TAAs (41). Oncolytic viruses are designed in such a way that they selectively replicate in tumor cells leading to their lysis without affecting normal cells. Therefore, they cannot be considered as TAA-encoding, DC-targeted therapeutic vaccines, and are not within the scope of this review. A comprehensive review on oncolytic viruses is provided elsewhere (42).

**Figure 1:**
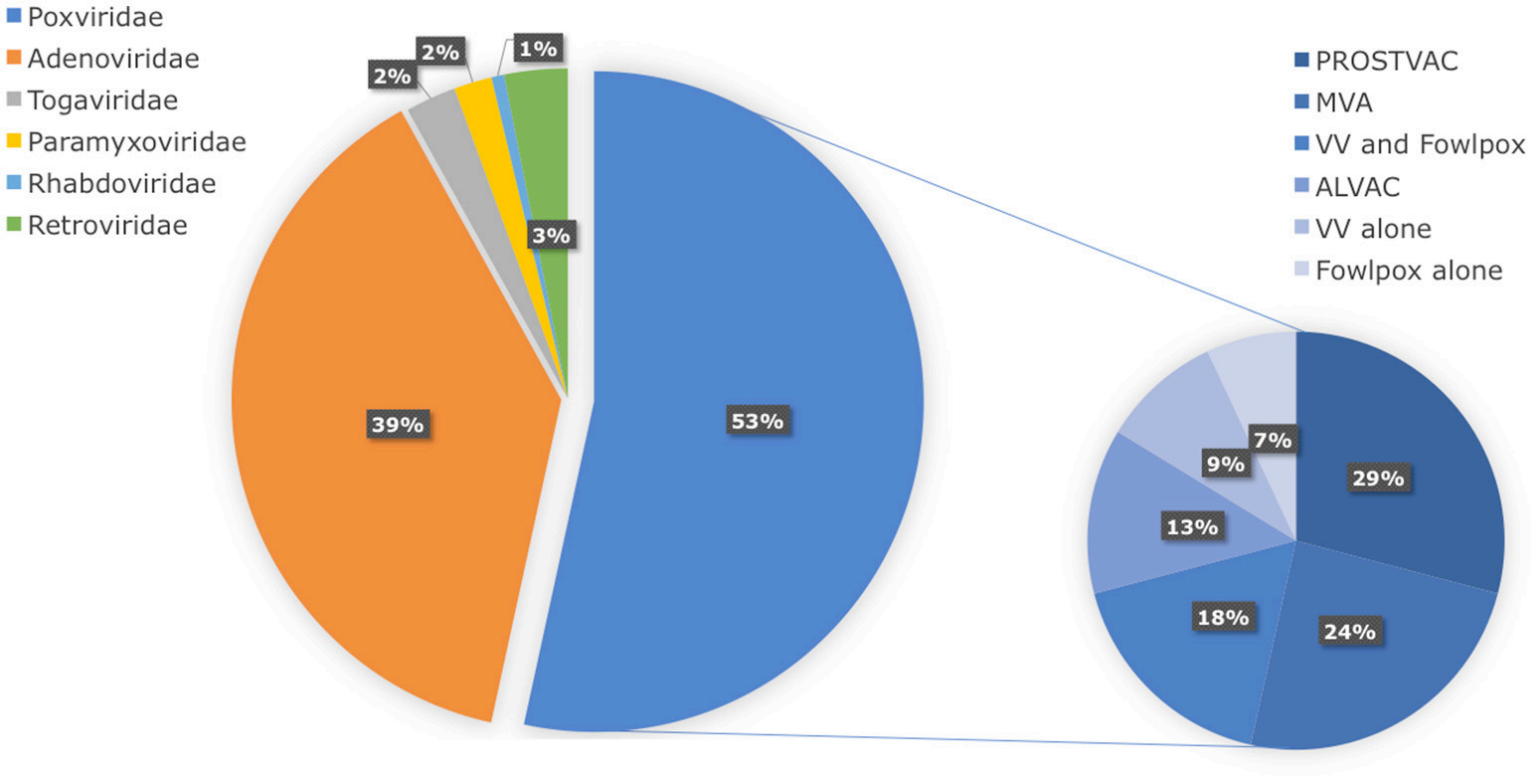
Distribution of viral vector families involved in ongoing or completed clinical trials. Within the search engine ClinicalTrials.gov from the National Institute of Health (NIH), the search terms “virus,” “cancer,” and “vaccine” yielded 325 search results, of which only 75 trials were selected based on the following criteria: *in situ* therapeutic viral vaccinations encoding TAAs with or without extra adjuvant. Oncolytic virus-based vaccines, preventive virus-based vaccines, virally modified DCs, tumor, or T cell-based vaccines were excluded.

In search of clinically relevant viral approaches to deliver TAAs to DCs *in situ*, we turned to “ClinicalTrials.gov.” As depicted in Figure 1, viral vectors derived from viruses of the *Poxviridae* family are most often used in clinical trials in the framework of antitumor immunotherapy with over 85 registered clinical trials. In comparison, less than 15 registered clinical trials involve therapeutic antitumor vaccination with viral vectors derived from viruses of the *Retroviridae, Togaviridae, Paramyxoviridae*, or *Rhabdoviridae* families. In this section we provide an overview of the journey these viral vectors made from the bench to the bedside.

### Viral vectors derived from viruses of the *Poxviridae* family

Poxviruses are enveloped dsDNA viruses with a linear genome that can infect mammalian cells. A major advantage of poxvirus-derived vectors is their ability to accept large inserts of foreign DNA and as such deliver large transgenes to target cells, including DCs (Table 2). Since viral replication and transcription occurs solely in the cytoplasm of host cells, the risk of insertional mutagenesis is precluded. By attenuating the viral system via deletion of certain pathogenic genes, the safety of poxvirus-derived vectors is enhanced, as this disables them to generate infective viral particles and complete their life cycle. This is exemplified by the recombinant vaccinia virus, which is based on the attenuated Wyeth strain. Another interesting asset is the fact that poxvirus-derived vectors are relatively easy to produce at high-titers and stability (43).

**Table 2:** Overview of clinical and preclinically tested viral vaccines for cancer.

	**Poxviridae**	**Adenoviridae**	**Retroviridae**	**Togaviridae**	**Rhabdoviridae**	**Paramyxoviridae**	**AAV**	**Coronaviridae**	**Papillomaviridae**	**Baculoviridae**
**Genomic material**	dsDNA	dsDNA	ssRNA	ssRNA	ssRNA	ssRNA	ssDNA	ssRNA	circular DNA	circular DNA
**Insert capacity**	>30 kb	< 7.5 kb (though HC-AdV 35 kb)	12 kb	8 kb	6 kb	6 kb	< 4 kb	6 kb	8 kb	>38 kb
**Production (titers)**	High	High	Moderate	High	High	Low	High	Low	Low to high	High
**Efficacy of tg delivery to DCs**	Broad tropism	Serotype dependent tropsm, infects dividing and non-dividing cells, transient expression	Psedotype dpendent tropism, infects dividing or/and non-dividing cells, stable integration with long term expression	Broad tropism with strong neuronal preference + high expression level	Broad tropism, highly transient expression	No	Serotype dependent tropsm, infects dividing and non-dividing cells, slow expression onset	DC-specific tropism	Epithelial tropism	Broad tropism
**DC stimulatory potential**	High	High	Wild type low, but attenuated vector high	Low but cytotoxic	Wild type low, but attenuated vector high	Induction of DC maturation	Moderate	High	High	High
**Pre-existing immunity**	High	High	Low	Low	Low	High	Moderate	Most likely high	Most likely high	Low (but serum complement inactivation)
**Biosafety level**	BSL-2	BSL-2	BSL2-3	BSL-1-2	BSL-2	BSL-1	BSL-1	BSL-2	BSL-2	BSL1-2
**Clinical phase as vaccine**	Phase I-III	Phase I-II	Phase I-II	Phase I-II	FDA-approved	Phase I	Not applicable	Not applicable	Not applicable	Not applicable

There are currently about 69 species divided over 28 genera described for this family. Humans, vertebrates and arthropods can serve as natural hosts. Vaccinia virus is the prototypical poxvirus that has been administered to roughly one billion people through the profoundly successful smallpox eradication program. The latter paved the way for its clinical evaluation as an anticancer vaccine. Accordingly, extensive evaluation of therapeutic vaccination with live recombinant vaccinia virus encoding TAAs such as carcino-embryonic antigen (CEA) or prostate specific antigen (PSA) started more than 20 years ago. For example, recombinant vaccinia virus expressing CEA or PSA (rV-CEA or rV-PSA) was administered to advanced carcinoma or metastatic androgen independent prostate cancer patients, respectively. This induced elevated levels of anti-TAA antibodies next to TAA-specific CTLs, capable of lysing TAA-expressing tumor cells *in vitro* (44, 45). Despite these immunologic occurrences, a lack of clinical response with tumor regression in most patients was observed. This may be explained by inadequate clonal expansion and/or cytotoxicity *in vivo* next to low antibody titers with low affinity (44, 46). Importantly though, as long as 10^7^ plaque forming units (PFU) were injected, no significant treatment-related toxicities were observed, apart from injection site reactions such as erythema and pustule formation in all patients, who were previously vaccinated against smallpox.

These results were in marked contrast with the preclinical evaluations of TAA-expressing recombinant viral vaccines showing significant anticancer activity in animal models. Suggested reasons for the marginal clinical effects are the intrinsic tolerance of the TAA in humans and the immunosuppressive effects of the tumor and its microenvironment. Furthermore “epitope dominance” of viral antigens over TAAs could derivate the immunological focus from the cancer cells toward the viral vectors themselves. A phenomenon that was reinforced by the observation that rV-CEA or -PSA could only be administered once, at most twice, to result in a measurable immune response as after the third injection, viral vector-neutralizing antibodies completely diminished the cellular and/or humoral anti-TAA effect. The search for alternatives that had less compunction with pre-existing immunity led to evaluation of two Avipoxviral strains namely canarypox (ALVAC) and fowlpox. Furthermore, an attenuated strain of vaccinia, named modified vaccinia Ankara (MVA) was generated via repeated passaging (>350 times) in chicken embryo fibroblasts. Interestingly, ALVAC, fowlpox and MVA can infect but not replicate in mammalian cells. This increases the overall patient safety, while ensuring TAA-expression for up to 3 weeks after infection before cell death is induced within the virally infected cells.

Since clinical responses with replication-deficient poxviral vectors was also marginal and repeated vaccination still suffered from viral epitope dominance, it was suggested to use prime-boost regimens to increase the therapeutic outcome. These regimens generally consist of at least two different consecutively administered poxviral strains expressing the same TAA. In an attempt to determine which prime-boost regimen to use, a small randomized trial compared rV-CEA as the initial priming vaccination with three ALVAC-CEA injections (VAAA), vs. three vaccinations with ALVAC-CEA, followed by one rV-CEA (AAAV) (47). The IFN production by T cells in response to CEA peptide was much higher in the VAAA arm than the AAAV arm, which was furthermore correlated with a striking difference in overall survival of five vs. zero patients out of nine respectively. This and other studies suggested that optimal usage of poxviral vaccinations is done by priming with recombinant vaccinia, followed by booster vaccinations with recombinant non-replicating vaccines and/or vectors. One of the most applied poxviral vaccines (>25 clinical trials) is represented by the PSA-encoding PROSTVAC, which is most often delivered via a prime-boost regimen consisting of recombinant vaccinia followed by fowlpox virus injection.

As outlined in the first chapter of this review, DCs are the main drivers of immunity and as such represent the leading targets in vaccination. Since several DC subtypes with different maturation and polarization states co-exist *in situ*, the induction of a T_H_1 polarized antitumor CTL response requires their proper stimulation. However, direct injection of a TAA-encoding viral vaccine can result in the infection of both APCs and non-APCs. In the latter case, TAAs will be expressed *via* MHC-I by the infected cells and only via MHC-II by an APC, if the infected non-APC released TAAs upon cell death or via secretion. Only when the viral vaccine directly infects DCs, processed TAAs will be abundantly presented via MHC-I and MHC-II together with the appropriate co-stimulatory molecules to initiate a cytotoxic T_H_1-supported CTL response. Especially if a MHC-II targeting signal, such as invariant chain (Ii) or LAMP-1/2, and/or cross-presenting stimulators, such as calreticulin or the non-hemolytic part of the Listeria monocytogenes virulence factor, listeriolysin O, are co-delivered (48). Injection of mice, bearing Human Papilloma Virus (HPV)-16 immortalized tumor, with vaccinia encoding E7 fused to listeriolysin O or LAMP-1, resulted in enhanced uptake and presentation *via* MHC-I, or MHC-I and MHC-II, respectively. What's more, tumors appeared to regress because of increased amounts of IFN-γ and TNF-α secreting CTLs within the spleen. Of note, only the vaccines with MHC-I directing listeriolysin O resulted in high intratumoral CTL infiltration as well (45).

Due to the abiding relatively weak clinical response rates, viral vaccines were pimped with co-stimulatory signals to skew a T_H_1 climate. Multigene constructs were generated that included both a TAA as well as one or more co-stimulatory genes such as CD80 (B7.1) or CD154 (CD40L) that could aid in the stimulation of DCs *in situ* and as such in the proper stimulation of TAA-specific CTLs. Building on promising preclinical data, ALVAC–CEA–B7.1 was injected intramuscularly into patients with advanced, unresectable CEA-expressing malignancies. The virus could induce CEA-specific peripheral blood T cells in a proportion of patients, and 3 out of 16 patients demonstrated transient disease stabilization, but no disease regression (49). Interestingly, preclinical efficacy of MVA was mainly attributed to CD4^+^ T cells and polyclonal h5T4-specific antibodies, as only weak CD8^+^ T cell responses were induced (50). Therefore, the addition of stimulatory immune checkpoints like inclusion of CD70 or mGITRL-fusion proteins has been tested preclinically to enhance CTL responses (51). More robust tumor regression with improved overall survival was reported when using viral vectors encoding mGITRL-fusion proteins. This was linked to stimulation of strong antitumor CTL-responses and depletion of FoxP3^+^ regulatory T cells (Tregs) (52).

Current observations point out in favor of adding several co-stimulatory molecules in one vaccine. The MVA-based cancer vaccine TG4010 targeting the MUC1 antigen has been tested in a phase II trial for renal cell carcinoma (37 patients, metastatic) combined with IFNα2a and IL-2. Though no objective clinical responses were observed in the form of complete or partial tumor regression, improved overall survival was demonstrated. Antivaccine and antiIL-2 antibodies, CD4^+^ T cells, and MUC1-specific CTL responses were reported. Importantly, patients that had MUC1-specific CTLs showed a longer survival compared to the overall population (53). Also, several clinical-grade poxviral vaccination approaches such as PROSTVAC and ALVAC are regularly tested with the inclusion of a triad of immune enhancing co-stimulatory molecules, namely CD80 (B7.1), CD54 (intercellular adhesion molecule-1 or ICAM-1), and CD58 (leukocyte function-associated antigen- 3 or LFA3), collectively designated as TRICOM. When this formula was used to vaccinate mice, superior TAA-specific responses were described compared to constructs that only contained one or two of these molecules (54). A vaccinia prime–fowlpox boost regime encoding two TAAs (CEA and MUC1) for the treatment of pancreatic cancer, termed PANVAC, has also been evaluated alongside TRICOM. Phase II results have been promising with increased median survival in those patients with a pre-trial life expectancy of 3 months. However, a phase III trial did not demonstrate any survival benefit. More encouragingly, two different studies enrolling patients with metastatic ovarian or breast cancer, showed TAA-specific immunity after administration of a CEA-MUC-1-TRICOM poxviral-based vaccine (55, 56). This immunity did result in stable breast cancer disease (5/13), tumor shrinkage (1/13) and even one complete response with a significant drop in serum IL-6 and IL-8.

Interestingly, poxviruses have also been injected intratumorally to bring TAAs and co-stimulatory signals in close proximity. When melanoma lesions were injected with a recombinant vaccinia virus expressing TRICOM, clinical responses were shown in more than 30% of patients (57). Furthermore, when a vaccinia-based vaccine encoding both PSA and TRICOM was injected intratumorally in 21 patients with locally recurrent prostate cancer, higher numbers of tumor-infiltrating CD4^+^ and CD8^+^ T cells could be demonstrated. Furthermore, local Treg function was reduced and up to 76% of patients had stable or improved serum PSA levels (58). Finally, ALVAC has also been tested as an intratumorally delivered adjuvant by combining ALVAC encoding human CD80 with ALVAC encoding human IL-12 in patients with surgically incurable melanoma. Fourteen patients received intratumoral injections on days 1, 4, 8, and 11. Unexpectedly, tumors injected with ALVAC-B7.1 and ALVAC-IL-12 showed higher intratumoral levels of immunosuppressive cytokines like IL-10 and VEGF, and decreased intratumoral levels of pro-inflammatory cytokines IL-12 and IFN-γ, when compared to tumors injected with saline. While no tumor regression was observed, all patients did develop neutralizing antibodies against ALVAC, suggesting that pro-inflammatory intratumoral strategies can also lead to the induction of negative feedback mechanisms that aggravate the immunosuppressive tumor climate (59).

In addition to co-stimulatory molecules, adjuvant or growth factors such as GM-CSF have been added to increase the targetable DC load. This approach was shown to induce local and systemic tumor immunity with effective clinical responses. To exemplify, in a randomized study with PROSTVAC and GM-CSF, or empty viral vector and saline injections, primary objectives of improved progression-free survival were not reached. However, an increased median overall survival compared with control subjects was reported (25.1 vs. 16.6 months; *P* = 0.015) (60, 61). Also when ALVAC-CEA with CD80 was compared to its combination with the adjuvant GM-CSF, disease stabilization was seen in 26% compared to 37% of patients, who received the combination (62).

Next to co-stimulatory cytokines and growth factors, a few trials with poxviral vaccines evaluated its combinatorial potential with other anticancer treatments, such as targeted therapy, chemo- or radiotherapy. A large randomized phase III trial involving 733 patients with metastatic renal cancer was conducted using MVA-5T4 in combination with first-line treatment of receptor tyrosine kinase inhibitor sunitinib, IL-2 or IFN-α. No overall survival benefit was seen in the vaccine arm. However, analysis in this larger trial did reveal a significant correlation between the magnitude of 5T4-specific antibody responses and improved patient survival (63). In contrast, a phase II trial of TG4010 combined with first-line chemotherapy (cisplatin plus gemcitabine) in advanced non-small cell lung cancer (NSCLC) demonstrated a significant 6 month increase in median survival (64). It was recently shown in a randomized phase II study with 220 NSCLC patients that the combination of TG4010 with several chemotherapy regimens led to responses against MUC1, which correlated with improved survival under TG4010 treatment. Furthermore, these responses were associated with CTL responses against non-vaccine TAAs, thus evidencing epitope spreading (65). Finally, recombinant vaccinia virus encoding the HPV16 and 18 E6 and E7 fusion protein, was evaluated with heat shock protein 70 (HSP70) encoding DNA and TLR7-stimulating imiquimod. This led to a potent antigen-directed antibody and cytotoxic response in a phase I/II clinical trial for patients with (pre-)malignant cervical lesions (66–68). Since the arrival of antagonistic checkpoint inhibitor therapies, also their combinatorial potential with poxviral vaccination has been tested in metastatic castration-resistant prostate cancer. No dose limiting effects were observed while 58% of the chemotherapy naïve patients had a PSA decline from baseline (69).

Despite the growing use of poxviral vectors as antitumor vaccine candidates for cancers encoding a diverse range of TAAs such as CEA, PSA, MUC1, NY-ESO, Epstein Barr Virus nuclear antigen-1 (EBNA1), latent membrane protein-2 antigens (LMP-2), 5T4, melanoma antigen recognized by T cells-1 (MART-1), gp100, tyrosinase, HPV16 and 18 E6 and E7; their innate stimulatory properties remain poorly characterized. Interestingly, when the innate immune profiles elicited by ALVAC, MVA, and New York vaccinia virus (NYVAC) were compared *in vivo* in rhesus monkeys and *in vitro* in human peripheral blood mononuclear cells (PBMC), they appeared to be all distinct. ALVAC elicited a higher induction of proinflammatory and IFN-related antiviral cytokines with chemokines on day 1 following immunization. In addition, ALVAC's stimulatory phenotype was influenced by several PBMC subsets such as T cells, monocytes, macrophages, and pDC. Furthermore, the stimulatory phenotypes observed following priming with ALVAC, MVA, or NYVAC were all reduced when these poxviral vectors were used as a boost (70). Interestingly, Hanwell et al., compared TAA-expression and immunogenicity of 5T4 or gp100 delivered by ALVAC or MVA (71). While 5T4 expression in chicken embryo fibroblasts was equal for both vector systems, ALVAC-derived gp100 was much faster degraded compared to MVA-derived gp100. Furthermore, the HLA-A2 transgenic mouse model was used to measure CTL-responses upon vaccination. It was shown that vectors encoding 5T4 elicited low to immeasurable responses irrespective of the virus strain used. In contrast, MVA-vectors encoding gp100 elicited a significantly higher gp100-specific response than ALVAC-vectors encoding gp100, reflecting the *in vitro* TAA expression and stability (72). The above studies confirm the complexity of the possible immunological outcomes that depend on immunogenicity of the vector as well as the transgene it encodes, *in vivo* stability of transgene expression and order of vaccination in prime-boost regimens. Additional studies are required to evaluate the correlation between these different innate signatures, subsequent adaptive immune responses, and protective efficacy.

### Viral vectors derived from viruses of the *Adenoviridae* family

Adenoviruses are non-enveloped dsDNA viruses efficient at delivering DNA to both dividing and quiescent cells, like DCs. Furthermore, they can be readily produced with high titers up to 10^9^ IFU/ml that can be concentrated to 10^13^ IFU/ml (43). Early cancer vaccination studies used replication-incompetent variants (deletions in E1 and E3 region) of serotypes Ad2 and Ad5 encoding a range of TAAs. However, most humans show pre-existing immunity against these viruses, as a result of lifelong exposure to the wild type virus, especially against the most common serotype (Ad5). This hampers therapeutic efficacy through induction of neutralizing antiviral antibodies and/or CTL-mediated immunity, and moreover entails the risk of toxicity upon systemic adenoviral vector administration. In search for safer adenoviral vectors, a third generation high capacity HC-AdV, stripped of all viral coding sequences was engineered (73). Consequently, this HC-AdV is less immunogenic. Furthermore, this HC-Adc has a larger packaging capacity of up to 35 kb. From the adenoviral vector trials related to DC activation *in situ*, about 50% of the trials use TAA-encoding vaccines, while the other 50% only encode pro-inflammatory factors such as IL-12, type I or type II IFN, TNF-α, Flt3L, *et cetera* or co-stimulatory molecules such as CD40L.

Preclinical testing of various adenovirus-based antitumor vaccines demonstrates the induction of both protective humoral and cellular immunity as well as eradication of established tumors in mice (74–83). When different routes of administration were compared, intravenous and intradermal delivery appeared the most efficacious for antitumor immunity (79). Though preclinical animal models often respond well to vaccination, more variable vaccine responses are elicited in cancer patients with little therapeutic benefit (41, 84, 85). A phase I study for metastatic melanoma, showed that Ad2 encoding MART-1 (*n* = 36) or gp100 (*n* = 18), were safe, but failed to induce immunological or clinical efficacy (86). Remarkably, in one patient receiving the Ad2-MART-1 vaccine, a complete response was observed that could be attributed to the vaccination (86). One way to decrease vector neutralizing antibodies was by delivering a heterologous prime-boost. While only 50% of patients receiving naked DNA encoding CD86 and prostate-specific membrane antigen (PSMA) showed signs of successful immunization, this was 100% when they were inoculated with 5 × 10^8^ PFUs of PSMA-encoding viral vectors followed by PSMA plasmid boosts (87). On the other hand, when 13 NSCLC patients received sequential DNA and adenoviral vaccines coding for the lung tumor antigen L523S intramuscularly, this only resulted in L523S-specific sero-reactivity in one patient (88).

Pre-existing immunity to the adenoviral serotypes might be explanatory for their variable efficacy. This is supported by studies designed to circumvent antibody-mediated neutralization such as the *ex vivo* approach, i.e., infecting DCs and using these as a cellular vaccine. In one such study, advanced melanoma patients received DCs transduced with adenoviral vaccines encoding MART-1 and gp100. While one out of 17 patients experienced a complete response, three developed post-vaccination vitiligo. The latter signifies the generation of antigen-specific immunity that was even able to break tolerance to self-antigens (89, 90). In another phase I/II study, metastatic melanoma patients received three intradermal injections of adenoviral transduced DCs. Vaccination-induced CD8^+^ and CD4^+^ T cell responses to MART-1 were found in 6/11 and 2/4 evaluable patients, respectively. Evidence of epitope spreading was obtained in two patients, implying that the elicited T cells showed strong tumor reactivity. Out of the 14 patients receiving all three vaccines, one was considered tumor free, four had durable stable disease, and one remained disease-free after becoming eligible for a surgical resection (91). This positive outcome is not limited to highly immunogenic melanoma. A phase I trial was also performed in NSCLC patients, showing success in individual cases. Patients received multiple vaccines of DCs transduced with p53 encoding adenoviral vectors, 28% of patients demonstrated partial tumor regression or stable disease (92). Recently, a multi-genetically modified DC vaccine was generated based on an adenovirus that delivered two different TAAs (survivin and MUC1), the TLR5 agonist flagellin for DC maturation and a RNA interference moiety to silence the intracellular immune checkpoint molecule SOCS1. This vaccine was found to be safe and induce a complete remission rate of 83% in a phase I trial with 12 acute myeloid leukemia patients (93).

In conclusion adenoviral vaccines are mainly evaluated for *ex vivo* modification of DCs since pre-existing immunity hampers repeated injections *in vivo*. Whether *in situ* targeting of DCs with next-generation adenoviral vectors can lead to tumor regression, remains to be evaluated.

### Viral vectors derived from viruses of the *Retroviridae* family

All members of the *Retroviridae* are characterized by a ssRNA genome that is reverse transcribed into pro-viral DNA in the cytoplasm of the infected host cell. Subsequently this pro-viral DNA is inserted in the host cell genome, leading to permanent gene transfer. This asset makes retroviruses ideal blue prints for development of gene therapy vectors as they permanently modify the target cell of choice (94). Two genera within the *Retroviridae* family are most commonly applied namely the γ-retroviruses and the lentiviruses. While most members of the *Retroviridae* only replicate in dividing cells, lentiviruses uniquely replicate in non-dividing cells. However, lentiviral vectors (LVs) are not very efficient at transducing DCs as the reverse transcription process requires cellular deoxynucleoside triphosphates, which are extremely low in DCs. Interestingly, the addition of the lentiviral accessory protein Vpx to the LV is able to enhance their DC-specific infectivity by countering the low dNTP levels (95, 96). Furthermore LV transduction of DCs does not affect their immunophenotype, viability, or maturation capability while lack of pre-existing immunity allows repeated injections (25, 97).

However, the very first clinical trials performed with a γ-retrovirus-derived vector to successfully treat X-linked severe combined immunodeficiency, resulted in the development of leukemia in four out of nine children due to oncoretrovirus-mediated activation of the *LMO2* oncogene (98, 99). This unfortunate event created a major setback for the translation of vectors derived from the *Retroviridae* family to clinical applications. Though LVs are derived from a different genus and have a lower propensity for integrating in potentially dangerous regions within the human genome (100), these studies instigated the optimization of safer LV systems with engineered envelopes, pro-viral and/or packaging proteins (101–103). An additional safety feature comprises the mutation of the LV integrase, which impairs pro-viral integration into the host genome. Although this feature reduces the risk of insertional mutagenesis, non-integrative LV expression is less stable because it remains episomal and loses the transgenes after target cell replication, as with adenoviral vectors.

Despite the ample preclinical evidence that LVs represent safe and potent anticancer vaccines (25, 97, 104–107), their clinical use for this purpose remains low. Only in the field of adoptive transfer with *ex vivo* transduced chimeric antigen receptor T cells (CAR-T cells), LVs have taken a prominent place in cancer therapy with about 60 clinical trials registered today. The few active vaccination-related clinical trials involve subcutaneously delivered integrase-deficient LVs encoding NY-ESO-1. In addition, these are directly targeted to DCs *in vivo* through pseudotyping with a modified Sindbis virus envelope protein (DC-SIGN) and are termed LV305 (108). Preclinical murine models showed that the LV305 could be injected more than three times to recall peak-levels of CTLs. Furthermore, biodistribution appeared to be limited to the site of injection and draining lymph node with therapeutic efficacy in tumor bearing mice. Currently LV305 is being evaluated in phase I and II clinical trials for advanced, relapsing or metastatic solid tumors that express NY-ESO-1 such as melanoma, sarcoma, ovarian cancer, and small cell lung cancer. The vaccine is either being used as a single agent or in combination with other cancer drugs. These other drugs include anti-programmed death 1 (PD-1) therapy (pembrolizumab). So far, the first female patient with metastatic and recurrent synovial sarcoma, induced a robust NY-ESO-1-specific T cell response after three injections of LV305 with subsequent disease regression of 85% over 2.5 years (109). Furthermore, intradermal LV305 together with intramuscular delivery of G305 is studied as a combination product termed the CMB305 vaccine regimen for the treatment of sarcoma. G305 comprises a NY-ESO-1 recombinant protein and a TLR4 triggering glucopyranosyl lipid adjuvant stable emulsion (GLA-SE), with potential synergistic immunostimulatory and antineoplastic activities. So far, the vaccine regimen was well tolerated and generated a strong anti-NY-ESO-1 specific immune response in more than 50% of sarcoma patients with significant growth arrest and an overall survival rate (110). In general, CMB305 results in stronger and broader integrated responses than LV305 alone, underpinning the potential of heterologous prime-boost regimens. Finally, a fully enrolled, open-label, randomized phase II study is currently evaluating the safety and efficacy of CMB305 in combination with anti-PD-L1 therapy (atezolizumab) in 88 patients with advanced sarcoma. So far, patients receiving the combination experienced greater clinical benefit, more robust immunity and improved overall survival compared to atezolizumab alone.

### Viral vectors derived from viruses of the *Togaviridae* family

*Togaviridae* comprises alphaviruses which are small enveloped viruses that transfer a self-replicating ssRNA genome (111). Advantages of alphaviruses for therapeutic vaccination are their high-level expression of encoded proteins due to genomic replication next to lack of pre-existing immunity. Additionally, high-titer virus production is achieved in less than 2 days, be it at a high cost. Their strong preference for expression in neuronal cells has made alphaviruses particularly useful in neurobiological studies (112). In general alphavirus-based vectors are replication-deficient and require a helper vector for packaging of recombinant particles (113). Semliki Forest virus (SFV), Venezuelan Equine Encephalitis (VEE) and Sindbis virus have all been engineered as efficient replication-deficient or -competent vectors. Moreover, variants of the Sindbis virus have been preclinically explored for their differential abilities to target and activate DCs *in vitro* and *in vivo* (114). Importantly, human and mouse DCs were differentially infected by selected variants, suggesting differences in receptor expression between human and murine DCs. Despite these results, only the SFV and VEE have been tested clinically for their potential to engineer DCs *in situ*.

The SFV is an insect alphavirus that is able to infect dividing and non-dividing cells. A replication-incompetent SFV-based vector encoding the HPV derived antigens E6 and E7 has been evaluated preclinically (115, 116). This vector is currently tested in a phase I clinical trial for the treatment of (pre)-malignant cervical lesions (Vvax001). Furthermore, this replication-defective SFV-vector has been evaluated as an IL-12 encoding adjuvant that is encapsulated in cationic liposomes (LSFV-IL-12). This encapsulation approach tends to passively target the LSFV-IL-12 to tumors and enables repeated administration without the generation of antiviral immunity. The safety of administering these SFV-based vectors intravenously was shown in a phase I clinical study in melanoma and renal cell carcinoma patients. In addition, this LSFV-IL-12 has been described in a phase I/II protocol for the treatment of glioblastoma multiforme in which the vaccine will be infused intratumorally (117).

Secondly, virus-like replicons have been generated from an attenuated strain of VEE with potential antineoplastic activity (118, 119, 120). This self-amplifying replicon was evaluated in a phase I clinical trial for its safety and efficacy to deliver HER2 and is termed AVX901 (121). More specifically 22 patients with HER2-overexpressing (breast) cancer were evaluated, alone or in combination with other HER2-targeted therapies such as trastuzumab. Importantly, early clinical data did not report any dose-limiting toxicities, supporting the safety of this vaccine. In addition, two trials with the same virus-like replicon, but then encoding CEA termed AVX701, are registered for the treatment of colon and/or colorectal, breast, lung, and pancreatic cancers (122, 123). When the immune responses generated with AVX701 in colorectal cancer patients were compared between stage III and IV patients, the latter showed a trend for longer survival. In contrast, the antibody and T cell response tended to be higher in stage III patients, possibly reflecting a less immunosuppressive milieu in the latter.

The strong cytotoxic effect of alphavirus-based vectors on host cells, holds drawbacks for their use as anticancer vaccine moieties. In contrast, this feature is highly appreciated for oncolytic vectors as reflected in the amount of ongoing studies with oncolytic alphavirus-based vectors (124).

### Viral vectors derived from viruses of the *Rhabdoviridae* family

*Rhabdoviridae* are enveloped, bullet-shaped (rhabdos refers to rod) virions encapsulating ssRNA. In cancer therapy, this family is mainly known because of its oncolytic virus members derived among others from Vesicular Stomatitis Virus or Maraba virus (125, 126). In the framework of antitumor vaccination, this family is clinically represented by only one vaccine termed YS-ON-001. This is an inactivated rabies vaccine combined with TLR3-stimulating polyI:C for advanced solid malignancies. In 2016 and 2018, this was granted an orphan drug designation by the FDA for the treatment of hepatocellular carcinoma and pancreatic cancer, respectively (127, 128). The vaccine was shown to re-activate the suppressed tumor microenvironment with stimulation of T_H_1 cells, DCs, macrophages, B cells, CTLs and NK cells while downregulating Tregs. Currently also a phase I trial for the treatment of liver and breast cancer upon its intramuscular administration is ongoing.

### Viral vectors derived from viruses of the *Paramyxoviridae* family

*Paramyxoviridae* are represented by measles virus-derived vectors, which are enveloped ssRNA viruses that are mainly tested as oncolytic therapeutics (129). Confusingly, two clinical trials evaluated the therapeutic vaccination potential of oncolytic CEA-encoding vectors derived from the Edmonston measles strain (MV-CEA). Importantly, here CEA was not used as a TAA but to facilitate the *in vivo* monitoring of viral gene expression and replication (130). A first study (NCT00408590) started in 2004 with 37 participants for the treatment of ovarian epithelial cancer or primary peritoneal cancer. Intraperitoneal delivery of MV-CEA was well tolerated and resulted in stable disease for about 66% of patients. In 2006, the NCT00390299 trial was initiated to assess the safety and toxicity of intratumoral administration of MV-CEA for the treatment of recurrent glioblastoma multiforme (131). As this trial was suspended, no results have been disclosed so far.

The general consensus from published (pre-)clinical studies is that virus-based vaccines have the potential to be both safe and efficacious. Nevertheless, to raise the overall survival rates, further fine-tuning and clinical testing are imminent.

## Preclinical evaluation of novel viral vaccines

### Viral vectors derived from adeno-associated viruses (AAVs)

AAVs are small replication-defective non-enveloped ssDNA parvoviruses. They can only replicate inside the cell in the presence of a helper virus, such as adenovirus. However, AAV genomes can establish latency and persist as episomes in the absence of a helper virus or, in some rare cases, can even integrate into the host genome, particularly in a specific region of chromosome 19 (AAVS1). AAVs are able to infect dividing and non-dividing cells, making them attractive for delivery of transgenes to DCs. Moreover, they sustain long-term gene expression with low immunogenicity. These characteristics and their good safety profile make them appealing candidates for immunotherapy.

When an AAV vector containing the HPV16 E7 gene was used to infect mouse DCs, efficient gene transfer and DC activation was observed with upregulation of CD80 and CD83 next to T cell stimulation (132). Similarly, AAVs have been used to infect human DCs with HPV16 E7 (133), cytomegalovirus antigens (134), PSA (135), Her2/neu (136), or lactadherin, a membrane-associated self-glycoprotein that is expressed in breast cancer cells (137). Analogous to the observations with mouse DCs, efficient activation and priming of antigen-specific CTLs upon infection was observed. Furthermore, when an AAV-derived vector encoding HPV16 L1 protein, was used to immunize BALB/c mice intramuscularly, strong antibody titers were observed next to accumulation of APCs such as macrophages and DCs. In addition, the added benefit of co-vaccination with an adenovirus encoding murine GM-CSF was shown (138). Also the addition of a minimal CD11c promotor in the AAV expression cassette improved the infected DCs' ability to stimulate CTLs (139).

Even though AAVs are less immunogenic than adenoviral vectors, antibody neutralization due to previous exposure of the patient to multiple AAV serotypes, remains a common limitation for successful gene therapy and repeated vaccination (43, 140). Numerous AAV serotypes have been identified so far, with variable tropism depending on their route of administration (141). Therefore, an obvious approach to overcome neutralizing antibodies a specific AAV serotype is the use of a different serotype or naturally occurring AAV variant (142). To further enhance the outcome of AAV immunization, a rational design of its capsid can be performed by site-directed mutagenesis of surface-exposed serine and threonine residues. As such, a capsid-optimized AAV (serotype 6) showed a 5-fold increase in its transduction efficiency of bone-marrow derived DCs. In addition its intramuscular injection in prostate tumor bearing mice, resulted in PAP-specific CTL induction and tumor growth suppression (143). While these studies set the stage for clinical applications with capsid-optimized AAVs, the only clinical studies employing AAVs so far aim to use *ex vivo* AAV-modified DCs to expand CEA-specific CTLs present in blood of patients with grade IV gastric cancer and use these T cells for adoptive transfer (NCT01637805).

### Viral vectors derived from coronavirus

The enveloped coronaviral vectors carry a 31 kb autonomously replicating ssRNA genome and offer the advantage of being safe, since they do not create a DNA intermediate upon infection. Furthermore, they are able to exploit a diverse range of surface molecules to infect target cells. Some of them recognize the DC-specific C-type lectin DC-SIGN, which endows them with the ability to target DCs *in vitro* and *in vivo* (144). The group of Volker Thiel evidenced this with a biosafe coronavirus-based vector encoding human Melan-A with or without GM-CSF. In addition they reported that a single intravenous immunization with only 10^5^ PFU, resulted in a prophylactic and therapeutic immune response against metastatic melanoma (145). Furthermore, they also showed that human DCs, transduced with Melan-A-recombinant human coronavirus 229E, efficiently activated tumor-specific CTLs. That same group also demonstrated that vectors encoding Flt3L, exhibited a higher capacity to induce DC maturation compared to vectors delivering IL-2 or IL-15. The former more efficiently induced tumor-specific CTLs with expanded epitope repertoire, resulting in therapeutic tumor immunity (146).

The natural DC tropism combined with relative low doses needed, hold high potential for future clinical evaluation. However, as the *Coronoviridae* are believed to cause a significant amount of common colds in human adults, the risk of vaccination-limiting pre-existing immunity issues will need to be investigated.

### Viral vectors derived from papillomavirus

Papillomaviruses are small non-enveloped, circular dsDNA viruses. As widely accepted, chronic infection with certain HPV genotypes forms a major etiological factor for cervical cancer. For prophylactic vaccination, the HPV-derived capsid proteins L1 and L2 embedded in virus-like particles are profoundly exploited (147). For therapeutic vaccination, the oncogenic E6 and E7 antigens represent ideal targets because they are essential to the induction and maintenance of cellular transformation. Today several therapeutic vaccines for the treatment of HPV^+^ cervical malignancies are being investigated (148). However, when a prime/boost with an adenovirus type 5 vector was performed to a cervicovaginal model antigen, the high systemic CD8^+^ T cell response failed to induce intraepithelial CD103^+^ CTLs, necessary for protection against local challenge (149). These observations suggest that the epithelial tropism of HPV itself endows them with an interesting feature for their use as therapeutic vaccines. A major advantage of HPV as a viral vector system (HPV pseudovectors), is its capacity to package plasmids up to 8 kb in length, completely devoid of viral sequences (150). Upon an HPV intravaginal prime/boost with different HPV serotypes, a durable cervicovaginal antigen-specific CTL response was induced by promoting local proliferation and retention of primed CTLs (149).

### Viral vectors derived from *Baculoviridae*

The enveloped family of *Baculoviridae* has been preclinically evaluated to develop anticancer vaccines. This family forms an exception in the sense that they normally infect insects at larval stage. Hence since the 1940s, they have proven to be useful biopesticides in the field of agriculture (151). Furthermore, baculovirus-mediated expression of recombinant heterologous proteins in cultured insect and mammalian cells also represents a widely used and robust protein production method (152). Vaccination with the tumor-specific immunoglobin Id is considered a valuable approach for the treatment of lymphoma patients. Methods to improve its immunogenicity have been explored, leading to Id production via baculovirus-infected cells. Due to the addition of terminal mannose residues, typical for recombinant proteins expressed by insect cells, the Id proteins had enhanced immunostimulatory properties. Moreover, these Ids showed higher binding and activation capacity for human DCs next to higher elicitation of tumor-specific CTLs and eradication of pre-established murine lymphoma (153).

More recently, baculoviruses have been considered useful in gene therapy as well, as they (1) infect though not replicate in mammalian cells, (2) show low cytotoxicity, and (3) are able to carry large foreign genes into their 80–140 kb spanning genome (154). Baculovirus was shown to efficiently transduce and activate DCs *ex vivo* with upregulation of co-stimulatory molecules, MHC, type I IFN and other pro-inflammatory cytokines (155). Moreover, these DCs generated robust antitumor immunity in tumor bearing mice (154). Intradermal injection of wild type baculovirus (adjuvants) together with tumor cell lysates has also shown antitumor efficacy in several murine cancer models (156). Finally, a CEA-specific CD4^+^ T cell response was observed upon intramuscular injection of a CEA encoding baculovirus-derived vector (157).

Although there is no reported pre-existing anti-baculovirus immunity, these vectors could be highly immunogenic and as such rapidly inactivated by human serum complement upon systemic delivery (152, 158). Further preclinical studies are warranted though, their DC-transducing capacity, large gene insert capacity and biosafety profile represent promising features for future development of potent anticancer vaccines.

## Conclusions and future directions

While TAA-specific CTL responses are frequently induced upon vaccination with TAA-encoding viral vectors, most responses poorly translate into prolonged survival benefit for cancer patients (159, 160). The lack of overall clinical efficacy can be assigned to: (1) the fact that most patients received immunosuppressive (chemo)therapeutic regimens prior to vaccination, (2) pre-existing or induced vector-neutralizing antibodies, (3) lack of eligible TAAs, and (4) established tolerance to the TAA and linked herewith presence of a CTL suppressing tumor microenvironment.

The immunosuppressed status of heavily pretreated patients, as well as the immunosuppressive status of the tumor microenvironment, argues for the exploration of viral vaccines in earlier disease stages with less tumor burden. As the first virus-based vaccines have been approved by the FDA, their evaluation as early line treatments instead of last line becoming more likely. The immunogenicity of *in situ* administered viral vectors acts as a double-edged sword. The activation of DCs by viral vectors through recognition of pathogen-associated molecular patterns by pattern recognition receptors, such as TLRs, obviates the need for adjuvant (161, 162). Moreover, type I IFN-driven antiviral immunity is characterized by a T_H_1 response. Therefore, strong CTL responses are generated against TAAs that are delivered by viral vectors, as these are sensed as viral antigens. However, this immunogenicity entails that immunity is also build against viral components. This antiviral immunity precludes repeated injection of the viral vaccine, hampers prolonged transgene expression, neutralizes the vaccine and hinders the strength of TAA-specific cellular immunity (163, 164). Importantly, most of the clinically evaluated vectors like pox- and adenoviral vectors, show pre-existing immune responses in the host (165). A careful review of the literature on the topic of pre-existing immunity to viral vectors, suggests that this is indeed a hindrance. How pre-existing immunity impacts on the viral vaccine efficacy depends on the natural immunity to the vector. In essence all viral infections can elicit robust B and T cell memory responses (166), which can reduce antigen delivery by the viral vector due to neutralizing antibodies (167). Moreover, the pre-existing antiviral response will lead to rapid vector clearance and as such reduce exposure of the heterologous antigen (TAA) to the immune system. Finally, the immune response could focus on the strong viral antigens and “ignore” the co-expressed TAAs via the process of “epitope dominance.” Importantly, several approaches have been applied to avoid the downsides of pre-existing vector immunity, such as the use of vectors derived from non-human sources or from rare serotypes (83, 168). An alternative approach is provided by the “prime–boost” regimen in which two different recombinant viral vaccines expressing the same TAA are used consecutively (169). What's more, one can also alter the viral surface epitopes (envelope or capsid proteins) that might elicit neutralizing antibodies (170, 171). The inhibitory effect of pre-existing immunity can also be avoided by masking the viral vector inside DCs as discussed in the section on adenoviral-based vaccines (172). Besides, mucosal or high dose vaccination have also been shown to overcome pre-existing immunity problems (164, 173–175). A recent study showed that COX2 inhibitors, such as Celecoxib, can prevent the generation of neutralizing antibodies to vaccinia, allowing repeated administration without losing infectivity (176). Pre-existing immunity is however not an issue for all virus-based vaccines. For instance, the majority of the population has never been in contact with lentiviruses, making their vector derivatives attractive candidates for further vaccine development. Therefore, it may not be a surprise that the only lentiviral vaccine (LV305) that has been clinically evaluated in a handful of trials, all showed improved and durable responses in sarcoma patients (109, 110).

It should be noted that the route of administration profoundly affects the biodistribution of viral vectors, which can in turn influence their therapy efficacy and toxicity profile (43). While for example intravenous injection of AAVs via the tail vein triggers a CD4^+^ T cell-dependent humoral response, its delivery via the portal circulation leads to a T cell-independent B cell response (177). Importantly, while tissue-specific delivery can be an issue for naked protein or nucleic acid-based vaccines, viral vectors often hold a natural tropism for specific cells or tissues. As such, virus-based vaccines are excellent vehicles for tissue-specific delivery of transgenes together with its intrinsic immunogenicity. For example, adenoviral vectors are scavenged by the reticuloendothelial system after systemic injection, especially by Kupffer cells in the liver. However, upon intranasal administration of an IL-12 encoding adenoviral vector, pulmonary metastasis in a murine model of osteosarcoma could be treated without putative risks (178). As discussed, the epithelial tropism of the HPV-derived vectors themselves could endow them with the most optimal features for prophylactic and therapeutic HPV-related cancer vaccination. Additionally, some viral vectors have been extensively re-engineered in order the alter their tropism or transgene expression, as extensively discussed elsewhere (24). Targeting viral vectors to DCs has been explored as a means to tighten the control on where the viral vector is delivered to enhance the safety and efficacy. An approach that has been adapted to both lentiviral and adenoviral vectors is the use of single domain antibodies or so-called nanobodies that specifically bind APCs, albeit DCs or both DCs and macrophages (102, 179). Although it was expected that such an approach would enhance the vaccine efficacy, by avoiding presentation by non-professional APCs, this strategy did not deliver on its promise (180). This is in part explained by an enhanced anti-viral type I IFN response next to the lack of stromal cell transduction with reduced MHC-I mediated antigen presentation (181).

The ever-growing field of cancer antigen target identification should lead to a knowledge platform that can develop complete tumor eradicating vaccines. So far however, large clinical trials did not meet the expectations. This is most likely explained by the very inconsistent expression pattern of TAAs within the heterogenous tumor mass as well as their (vaccine-induced) tumor evasion over time (182, 183). The concept of neo-antigens harboring high-affinity T cell recognizable and tumor-unique epitopes, will become indispensable for the next generation antitumor viral vaccines. So far, mainly oncolytic viral systems have been linked to modulate the spectrum of neo-antigen specific CTLs with subsequent abrogation of systemic resistance to checkpoint inhibitor immunotherapy (184). Furthermore, both adenoviral and MVA vectors have been tested as neo-antigen encoding vaccines in the framework of human immunodeficiency virus related disease. More specifically, a genetic algorithm-based mosaic method was developed to generate artificial protein sequences that could increase the cross-reactivity of vaccine responses for diverse HIV-1 isolates. When these “mosaic” HIV sequences were delivered via adenovirus or MVA, this resulted in a strong protective effect against subsequent infection in non-human primates (185). These findings are encouraging for the development of cancer neo-antigen encoding viral vectors for the treatment of cancer.

Tumor-derived DCs are most often dysfunctional. As such they are less mature with low sensitivity to TLR activation, which is associated to STAT3 hyperactivity. Ideally, a vaccine should therefore consist of TAAs together with adjuvants to overcome the DCs' anergic state. While in the field of nanovaccines, several combinations have been explored (186), the delivery of more than one antigen/adjuvant/genetic silencer (e.g., small interfering RNA against STAT3) (187) is exactly what viral vectors could do. Especially viral vectors with a large genetic insert capacity such as poxvirus or baculovirus could be used for this purpose. Furthermore, viral vectors could also be used to target the delivery of proteins to cells of interest a.k.a. protein transfer vector or PTVs (188). Therefore, research into strategies to exploit the advantageous traits of viruses (e.g., high infectivity, adjuvant potential), while avoiding their traits developed to avoid immune responses (e.g., decreasing the translational machinery) should be continued.

**Figure 2:**
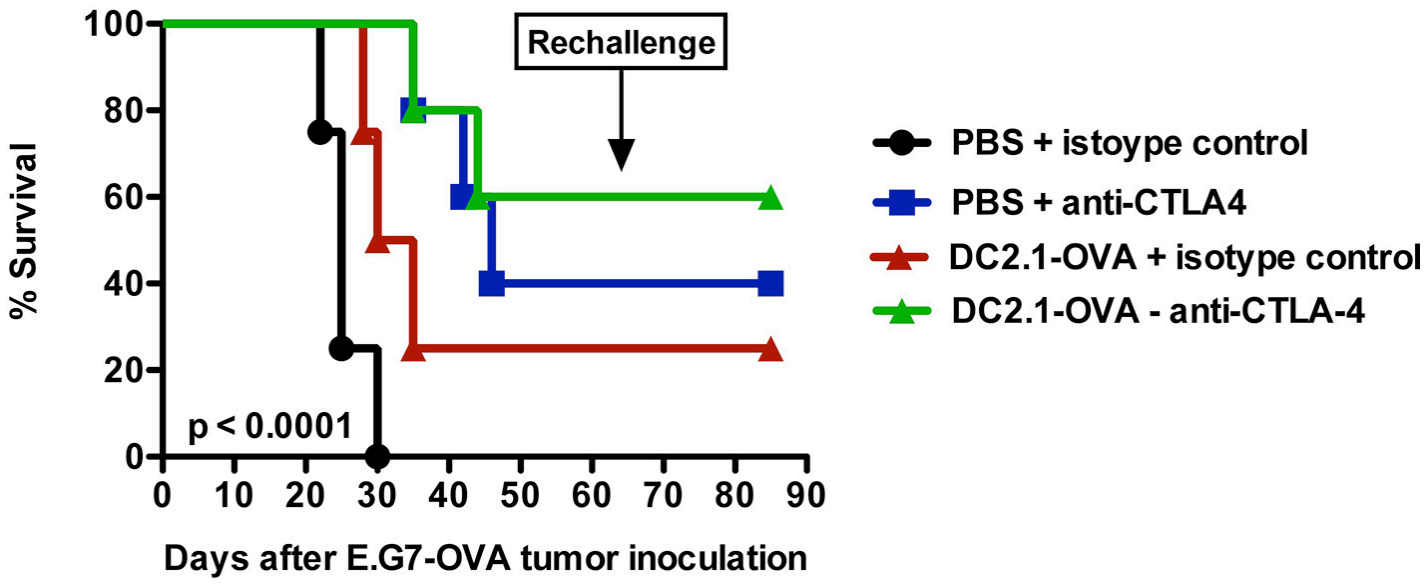
Intranodal vaccination of DC-targeted LVs in combination with anti-CTLA4 results in prolonged survival. To evaluate the therapeutic potential of DC-targeted LVs in combination with anti-CTLA4, C57BL/6 mice were challenged on day 0 with 3 × 10^5^ cells of an ovalbumin positive EL4 lymphoma line termed E.G7-OVA. Ten days later, mice were intranodally immunized with PBS or 10^6^ transducing units of single chain antibody or nanobody (Nb) DC2.1 pseudotyped LVs encoding OVA. Seven days later, the treatment was repeated. Furthermore, mice were treated on days 13 and 20 intraperitoneally with 50 μg isotype control or anti-CTLA4 antibody. Tumor growth and survival were examined every 2 days. The results shown are representative for one experiment with five mice per group.

Finally, it also makes sense to combine DC-targeted vaccines, purposed to elicit antitumor T cell responses, with strategies designed to support the function of T cells in the tumor microenvironment (148). In this regard immune checkpoint inhibitors might be ideal candidates. These drugs are able to release the brakes on T cells imposed by inhibitory receptors, such as CTLA-4 and PD-1. This is nicely exemplified by the combination of an adenoviral vector, encoding the murine breast TAA TWIST1, with intraperitoneal injection of a bifunctional anti-PD-L1/TGFβ fusion protein. This combination was shown to induce a more active CTL and NK cell phenotype within the tumor microenvironment (189). Previously, we performed a therapy experiment with the ovalbumin (OVA) expressing EL-4 thymoma model (E.G7-OVA) by combining a DC-targeted LV encoding OVA with anti-CTLA-4 treatment. This led to prolonged overall survival compared to the injection of LVs or anti-CTLA-4 antibodies alone (Figure 2). Moreover, this resulted in protection against a subsequent challenge with a lethal dose of E.G7-OVA cells, suggesting that DC-targeted LVs can be promising immunotherapeutics if combined with a T cell suppression counteracting strategy.

Nature has fine-tuned viruses to highly efficient gene transmitters in a cell-specific fashion with intrinsic adjuvant-like features. Hence an abundant range of viral vectors has been explored and tweaked substantially to develop anticancer vaccines with specific features. As a result we believe it will not be a matter of finding the “one-fits-all” vector but the “most appropriate combination” for the cancer type and stage at issue.

## Author contributions

All authors listed have made a substantial, direct and intellectual contribution to the work, and approved it for publication.

### Conflict of interest statement

The authors declare that the research was conducted in the absence of any commercial or financial relationships that could be construed as a potential conflict of interest.
